# Post-translational palmitoylation of metabolic proteins

**DOI:** 10.3389/fphys.2023.1122895

**Published:** 2023-02-24

**Authors:** Kaitlyn M. J. H. Dennis, Lisa C. Heather

**Affiliations:** Department of Physiology, Anatomy and Genetics, University of Oxford, Oxford, United Kingdom

**Keywords:** palmitoylation, membrane transporter, mitochondria, metabolism, fatty acid signalling

## Abstract

Numerous cellular proteins are post-translationally modified by addition of a lipid group to their structure, which dynamically influences the proteome by increasing hydrophobicity of proteins often impacting protein conformation, localization, stability, and binding affinity. These lipid modifications include myristoylation and palmitoylation. Palmitoylation involves a 16-carbon saturated fatty acyl chain being covalently linked to a cysteine thiol through a thioester bond. Palmitoylation is unique within this group of modifications, as the addition of the palmitoyl group is reversible and enzyme driven, rapidly affecting protein targeting, stability and subcellular trafficking. The palmitoylation reaction is catalyzed by a large family of Asp-His-His-Cys (DHHCs) motif-containing palmitoyl acyltransferases, while the reverse reaction is catalyzed by acyl-protein thioesterases (APTs), that remove the acyl chain. Palmitoyl-CoA serves an important dual purpose as it is not only a key metabolite fueling energy metabolism, but is also a substrate for this PTM. In this review, we discuss protein palmitoylation in regulating substrate metabolism, focusing on membrane transport proteins and kinases that participate in substrate uptake into the cell. We then explore the palmitoylation of mitochondrial proteins and the palmitoylation regulatory enzymes, a less explored field for potential lipid metabolic regulation.

## 1 Introduction

Protein lipidation dynamically governs many biological processes. The first published report of protein lipidation occurred in 1951 (Folch and Lees, 1951) and was later found to be regulated by enzymes that recognize lipids and their protein substrates. The chemical attachment of lipids to target proteins can be reversible or irreversible, and can affect protein function, structure, stability and localization. Both saturated and unsaturated lipids of varying chain length can attach to serine, cysteine, or lysine residues of proteins resulting in protein lipidation (Chen et al., 2018). Multiple lipid post-translational modifications have been identified including myristylation, farnesylation, gernaylgernaylation and S-palmitoylation [for full review see (Chamberlain and Shipston, 2015; Main and Fuller, 2022; Shang et al., 2022). Compared with other modifications, S-palmitoylation (referred to as palmitoylation) is distinguished by it being a readily reversible and dynamic reaction, and will be the focus of this review. Protein palmitoylation is a reversible post-translational lipid modification whereby a long chain fatty acid is covalently bound to a cysteine thiol through a thioester bond (Mitchell et al., 2006; Chamberlain and Shipston, 2015). The attachment of palmitate (C16:0) is the most common fatty acid present in palmitoylated proteins, although various other fatty acid moieties such as palmitoleate (C16:1), oleate (C18:1), and stearate (C18:0) can be used to a lesser extent (DeMar and Anderson, 1997; Muszbek et al., 1999; Liang et al., 2002). Due to the labile nature of thioester bonds, palmitoylated proteins can undergo cycles of palmitoylation and de-palmitoylation in response to extracellular stimuli, and have specific regulatory enzymes that influence these cycles. Compared to static lipid modifications, the unique reversibility of palmitoylation differentiates this PTM. In many cases, palmitoylation adds a hydrophobic membrane anchor, affecting membrane association (Kleuss and Krause, 2003). The reversible cycles of palmitoylation and depalmitoylation have been implicated in being a crucial component for protein trafficking between membrane compartments (Greaves et al., 2009; Jansen and Beaumelle, 2022). The timescale for these cycles ranges from seconds to hours (Rocks et al., 2010; Martin et al., 2012; Won and Martin, 2018).

Emerging evidence highlights palmitoylation as a potential regulator of cellular metabolism in various tissues. Plasma palmitate is imported into the cell across the plasma membrane, and once in the cytosol acyl-coA synthetase (ACS) catalyzes a thioester bond forming palmitoyl-CoA. Palmitoyl-CoA can also be synthesized endogenously from *de novo* lipogenesis from non-fatty acid precursors by fatty acid synthase (FASN). In addition to being a source of energy, palmitoyl-CoA is a biologically active signalling molecule that can regulate many physiological processes (Kerr et al., 2021). More specifically, through the attachment of palmitoyl-CoA derived from either exogenous FA uptake or from *de novo* FA synthesis (Qu et al., 2021), palmitoylation has been linked with substrate uptake into the cell through regulation of transport trafficking of specific membrane substrate transporters (Wang et al., 2019; Hao et al., 2020). More recently palmitoyl-CoA has been implicated in regulating various mitochondrial metabolic processes. This is of particular interest given that 95% of the total CoA pool is within the mitochondria (Idell-Wenger et al., 1978). Thus, palmitoylation sits at a key cross roads linking substrate supply of palmitoyl-CoA to regulation of substrate uptake and onwards metabolism *via* palmitoylation (Kim et al., 2019.; Qu et al., 2021). Therefore, this review will first discuss the role of palmitoylation in regulating glucose and lipid uptake across the plasma membrane through membrane transport proteins, which is an area of research that has received much attention of recent. We will then explore how palmitoylation is affecting mitochondrial metabolism, which represents a poorly understood mechanism of regulation.

## 2 Regulatory palmitoylation enzymes

Following the discovery of palmitoylation, understanding the mechanism by which palmitoyl-CoA is covalently attached to target proteins attracted great attention. An early report in 1996 observed that in the presence of palmitoyl-CoA, the G protein Giα1 was palmitoylated on Cys3, suggesting a spontaneous auto-palmitoylation mechanism-of-action relying on local palmitoyl-CoA concentrations (Duncan and Gilman, 1996; Smotrys and Linder, 2004). However, as physiological concentrations of palmitoyl-CoA are tightly regulated and following the initial discovery of regulated palmitoylation cycles (Wedegaertner and Bourne, 1994), pioneering studies in the early 2000s identified families of enzymes that regulate the palmitoylation reaction (Lobo et al., 2002; Roth et al., 2002, 2006).

### 2.1 DHHC palmitoyl transferases

Palmitoylation in mammals can be catalyzed by a family of 23 cysteine-rich zinc protein palmitoyltransferases (PATs) that are characterized by a conserved Asp-His-His-Cys (zDHHC) catalytic domain (Mitchell et al., 2006; Ohno et al., 2006). The zDHHC proteins have four to six transmembrane domains, and the conserved zDHHC cysteine-rich catalytic domain is located on the cytosolic face. It has been well established that the catalytic activity of zDHHCs is a two-step process involving palmitoyl-CoA. The first step involves auto-palmitoylation of the zDHHC enzymes, where the long chain acyl-CoA generates an acyl-enzyme intermediate. The fatty-acyl group is then transferred from the zDHHC to the target protein resulting in the palmitoylation of the target protein (Mitchell et al., 2010). Interestingly, zDHHCs display differing auto-palmitoylation capabilities and have different catalytic efficiencies (Iwanaga et al., 2009). An important feature of zDHHCs is their subcellular locations and tissue specificities. Many zDHHCs localize to the endomembrane compartments including the Golgi (e.g., zDHHC4, zDHHC8), endoplasmic reticulum (zDHHC1, zDHHC12) and plasma membrane (e.g., zDHHC5, zDHHC20), while others appear in multiple locations (Ohno et al., 2006; Rocks et al., 2010; Aicart-Ramos et al., 2011; De and Sadhukhan, 2018; Main and Fuller, 2022)]. Consequently, the differing subcellular locations of the DHHCs have been proposed to regulate varying, yet complimentary functions, and their synergistic efforts have recently been implicated in mediating protein trafficking between intracellular compartments (De and Sadhukhan, 2018; Wang et al., 2019).

While zDHHCs have been identified in various subcellular locations, there is a gap in our knowledge surrounding the presence of mitochondrial zDHHCs. As mentioned above, palmitoylation can occur enzymatically by zDHHCs or *via* spontaneous auto-palmitoylation (Duncan and Gilman, 1996; Veit et al., 1998; Bizzozero et al., 2001). Work by Kostiuk and others used a bio-orthogonal azido-palmitate probe to identify mitochondrial palmitoylated proteins in the mitochondrial matrix, and proposed that palmitoylation in this organelle may occur through non-enzymatic mechanisms (Kostiuk et al., 2008). While it still remains unknown if mitochondrial palmitoylation predominantly occurs enzymatically or non-enzymatically, zDHHC8 (Maynard et al., 2008) and zDHHC13 (Napoli et al., 2017; Shen et al., 2017) have been associated with mitochondrial processes. zDHHC13 deficient mice and knockdown cells displayed impaired mitochondrial function and abnormal lipid metabolism (Shen et al., 2017). Furthermore, loss of zDHHC13 from cortex and cerebellum in mice resulted in dysfunctional mitochondrial dynamics and altered metabolism through decreased Drp1 palmitoylation (Napoli et al., 2017). Although zDHHC8 and zDHHC13 modulate mitochondrial metabolic processes, the presence of zDHHCs within the mitochondria remains unclear. Interestingly, recent work by Pei and others identified zDHHC18 within the mitochondria in ovarian cancer cells (Pei et al., 2022). This is the first report to suggest zDHHCs are localized within the mitochondria and, as such, further investigations are warranted.

### 2.2 Depalmitoylating enzymes

As palmitoylation is well established as being a reversible process, the cleavage of palmitoyl-CoA from target proteins is imperative in maintaining the dynamic nature of the lipid PTM. Depalmitoylation occurs by enzymatic thioester bond hydrolysis by acyl thioesters (Long and Cravatt, 2011). Depalmitoylating enzymes in the α/β serine hydrolase family have been identified and localize mainly in the cytosol and lysosomes (Camp and Hofmann, 1993; Duncan and Gilman, 1998). The depalmitoylating enzymes include acyl protein thioesterase 1 (APT1/LYPLA1) (Duncan and Gilman, 1998), and acyl protein thioesterase 2 (APT2/LYPLA2) (Tomatis et al., 2010) palmitoyl-protein thiosterase 1 and 2 (PPT1, PPT2) (Camp and Hofmann, 1993; Soyombo and Hofmann, 1997) and α/β-hydrolase domain-containing proteins (ABHD) (Abrami et al., 2021). While little is known how depalmitoylating enzymes interact with membranes and subsequently catalyze the release of palmitoyl-CoA attached to target proteins, Abrami et al. (2021) recently described the mode of action of APT2. APT2 generates a weak membrane affinity *via* its β-tongue, and its membrane stabilization requires palmitoylation on Cys2 by zDHHC3 or zDHHC7 (Abrami et al., 2021). Upon finding a target protein located in the membrane, APT2 is able to extract the acyl chain into its hydrophobic pocket for hydrolysis (Abrami et al., 2021).

Similar to mitochondrial zDHHCs, less is known surrounding depalmitoylating enzymes associated with the mitochondria. Interestingly, APT1, which has historically been thought to only be cytosolic, has been identified in isolated mitochondria from humans and mice (Zhang et al., 2008.; Lefort et al., 2009). However, it was unknown whether APT1 was functionally active in hydrolyzing the thioester bonds of palmitoylated mitochondrial proteins. Using novel mitochondrial targeted S-deacylase probes (mitoDPPs) in live cells, Kathayat and others observed that the deacylase activity of APT1, but not APT2, was active in the mitochondria (Kathayat et al., 2018). Through immunostaining and subcellular fractionation experiments, they found that APT1 is primarily localized in the mitochondria (Kathayat et al., 2018), which is in contrast to previous reports using florescent protein tagging identifying APT1 trafficking mainly associated with the Golgi and plasma membrane (Kong et al., 2013; Vartak et al., 2014). It was also determined that APT1 activity was altered in response to mitochondrial lipid stress, revealing a potential regulatory mechanism of mitochondrial lipidation (Kathayat et al., 2018). While APT1 was observed in the mitochondria and contains depalmitoylase activity, the functional relevance of its presence remains unknown.

In addition to APT1, α/β hydrolase domain containing 10 (ABHD10), a related depalmitoylase from the serine hydrolyase family, was identified by proteomics to be located in the mitochondria (Li et al., 2010). This is in contrast to its other depalmitoylase family members ABHD17A, B and C, which are predicted to be located near the plasma membrane. ABHD10 has been implicated in regulating mitochondrial redox homeostasis through depalmitoylation (Cao et al., 2019). Knockdown of ABHD10 resulted in decreased cell viability and increased mitochondrial H_2_O_2_ levels under oxidative stress conditions (Cao et al., 2019). In contrast, ABDH10 overexpression improved cell viability and decreased H_2_O_2_ levels (Cao et al., 2019).

## 3 Membrane substrate transport proteins as targets for palmitoylation

The first step of cellular metabolism begins with substrate uptake across the plasma membrane. The plasma membrane encases the cell and is part of the extensive endomembrane system that includes the endoplasmic reticulum (ER), the nuclear membrane and the Golgi apparatus. Substrate uptake across the plasma membrane is a highly regulated process and often involves concerted efforts between specific membrane receptors and transport proteins. Substrates are transported across the plasma membrane by protein-mediated uptake (Kerr et al., 2017), which confers the physiological benefit of tightly regulating the amount of substrate uptake into the cell in response to metabolic demands, allowing for greater homeostatic control.

**FIGURE 1:**
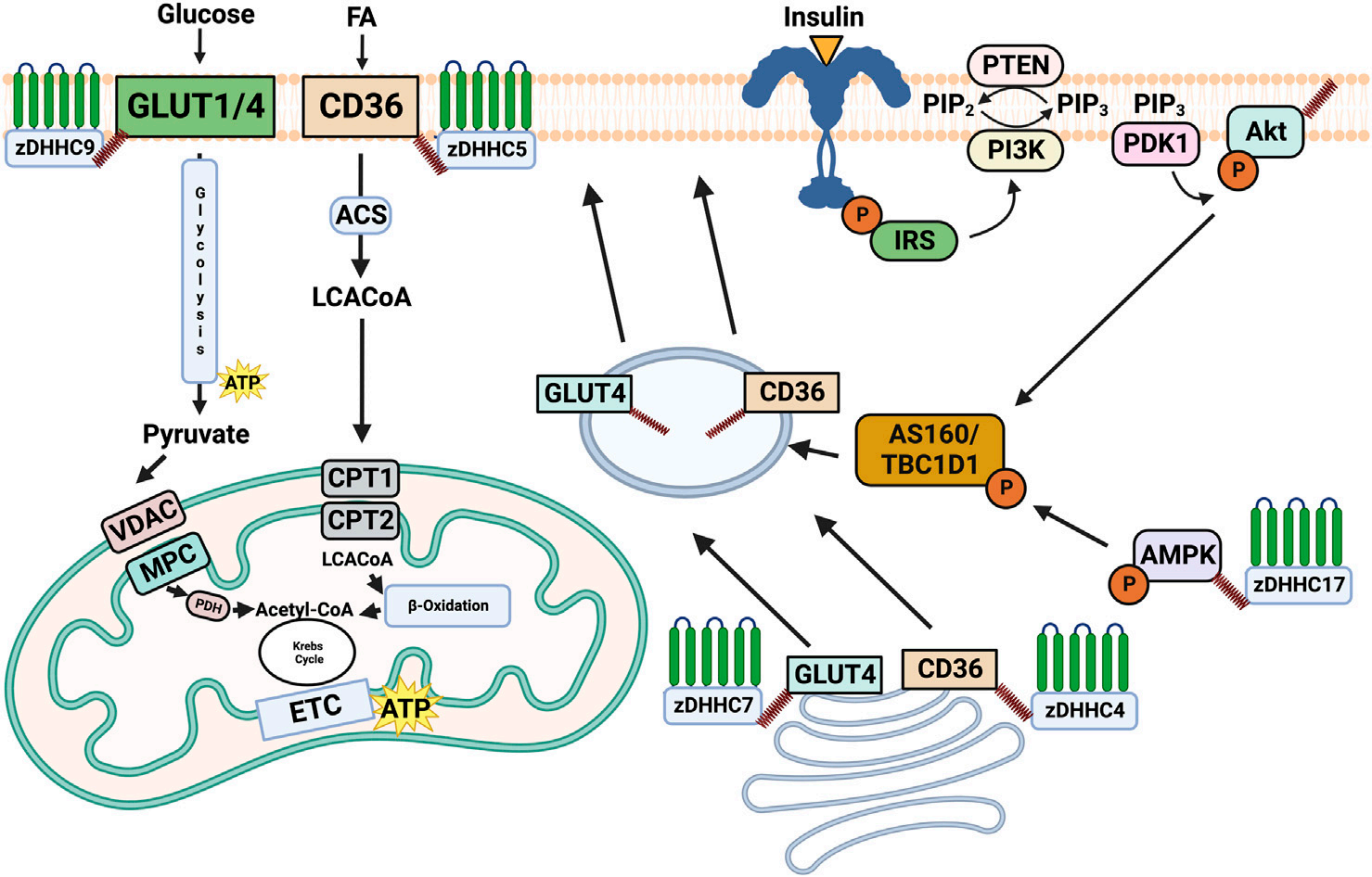
Overview of the role of palmitoylation in regulating substrate metabolism. Palmitoylation of CD36 and GLUT4 by DHHC4 and DHHC7, respectively induces their trafficking to the plasma membrane. Once on the plasma membrane DHHC5 palmitoylates and stabilizes CD36, while zDHHC9 palmitoylates GLUT1. Subcellular trafficking and maintenance on the plasma membrane driven by palmitoylation subsequently results in the uptake of fatty acids and glucose from CD36 and GLUT4/1. Palmitoylation of AMPKα by DHHC17 has been shown to be required for its phosphorylation and localization, while Akt palmitoylation by an unknown DHHC is required for its phosphorylation. While AMPKα and Akt are important modulators of substrate metabolism, the downstream consequences of their palmitoylation remain unknown. Made on biorender.com.

In response to cellular signals, specific membrane substrate transport proteins can translocate from intracellular depots to the plasma membrane, and the regulation of their trafficking and membrane association has emerged as a key mechanism underpinning metabolic control. To date, it has been shown that membrane transport proteins are heavily modulated through post-translational mechanisms and the link between these modifications and membrane trafficking is receiving increased attention. More specifically, the PTM palmitoylation has been proposed as a mediator of metabolic control in various tissues due to the addition of the hydrophobic palmitate tail to the substrate transport protein increasing its affinity for the membrane. We have outlined below some of the key plasma membrane transport proteins and two key kinases involved in the control of metabolism and explore their regulation through palmitoylation, which are outlined in Figure 1.

### 3.1 Palmitoylation of fatty acid transporter CD36

Import of FAs into various tissues is regulated by a family of plasma membrane FA transporters, including fatty acid translocase (CD36), plasma membrane fatty acid-binding protein (FABPpm) and fatty acid transport proteins (FATPs). Of the three main FA transporters, CD36 drives the majority of FA uptake (Luiken et al., 1999) and has been the only FA transporter to be identified as palmitoylated.

CD36 is a widely expressed and heavily glycosylated 88kD integral protein (Greenwalt et al., 1992). It is characterized by a hairpin membrane topology consisting of two transmembrane spanning regions with its NH_2_ and COOH termini located in the cytoplasm (Glatz et al., 2016). The outer ring of CD36 contains a large hydrophobic cavity that acts as a docking site for fatty acids and, through unknown mechanisms, promotes the transportation of FAs into the cell (Lisanti et al., 1994; Pohl et al., 2005; Pepino et al., 2014). CD36 has been well characterised to contain two phosphorylation sites, two ubiquitination sites and four palmitoylation sites, two on each of the NH2 and COOH cytoplasmic terminals of CD36 (Cys3, Cys7, Cys464, and Cys466) (Tao et al., 1996). In response to stimuli (i.e., AMP activated protein kinase or insulin), CD36 can rapidly and dynamically translocate from intracellular depots to the plasma membrane. Alterations in the amount of CD36 on the plasma membrane have been shown to regulate the fatty acid uptake rate (Glatz et al., 2016). As such, CD36 plays an important role in dynamically regulating fatty acid metabolism, and understanding the regulatory processes will provide mechanistic insight into lipid-derived regulation of metabolism.

Palmitoylation of CD36 was first discovered in adipocytes by Jochen and Hays (1993). Later work delineated that palmitoylation was involved in CD36 maturation and processing in the ER, and was necessary for subsequent trafficking to the Golgi (Thorne et al., 2010). Interestingly, following serine substitutions, all four of the cysteine residues on CD36 were found to be essential for insulin and AMPK induced translocation, confirming palmitoylation of CD36 is essential for translocation to the membrane (van Oort et al., 2014). DHHC4 and DHHC5 were identified as the palmitoytransferases responsible for CD36 palmitoylation and implicated in regulating its trafficking in adipocytes (Wang et al., 2019). Interestingly, these palmitoyltransferases are not functionally redundant. DHHC4 is important for CD36 palmitoylation in the endoplasmic reticulum to induce translocation to the plasma membrane, whereas DHHC5 palmitoylates CD36 at the plasma membrane and protects it from depalmitoylation (Wang et al., 2019). As such, it’s the coordination between the different localized DHHCs in cells that facilitates successful CD36 trafficking and maintenance at the plasma membrane (Wang et al., 2019). In adipocytes, deficiency of either DHHC4 or DHHC5 ablates fatty acid uptake. Furthermore, DHHC4 knock-out or DHHC5 adipose-specific knockout mice also display decreased fatty acid uptake (Wang et al., 2019). CD36 palmitoylation has been further implicated in regulating metabolism in the liver. Enhanced palmitoylation and increased localization to the plasma membrane was observed in mouse livers with non-alcoholic steatohepatitis (NASH) displaying abnormal lipid metabolism. Consequently, inhibiting CD36 palmitoylation was protective against the development of NASH (Zhao et al., 2018). Collectively, these studies highlight that CD36 palmitoylation, orchestrated by DHHC4 and DHHC5, is paramount in mediating FA uptake in adipose and liver tissue.

### 3.2 Palmitoylation of glucose transport proteins

Similar to FA uptake, the first and rate limiting step of glucose metabolism is transport across the plasma membrane. Glucose uptake is mediated by transport proteins of the GLUT family, and two isoforms, GLUT1 (glucose transporter 1) and GLUT4 (glucose transporter 4), are readily involved. GLUT4 and GLUT1 are facilitative transporters that transport glucose and other hexose sugars through their aqueous pores down a concentration gradient into the cell. The topology of the GLUT transporters was first reported for GLUT1 (Hruz and Mueckler, 2001), and GLUT4 is predicted to have similar structure. GLUTs are comprised of 12 transmembrane domains with a cytoplasmic and exofacial loop. These domains have hydrophobic parts facing the plasma membrane and the hydrophilic parts form the pore. The C- and N- terminus of the GLUT transporters are located on the cytosolic side of the membrane. GLUT1 is primarily located on the plasma membrane to facilitate basal glucose uptake, while GLUT4 is located in intracellular compartments under basal conditions and translocates to the plasma membrane in response to stimuli (i.e., insulin and contraction). Both GLUT1 and GLUT4 have been shown to be regulated by various PTMs, including palmitoylation.

#### 3.2.1 GLUT4

Under basal conditions, GLUT4 is sequestered on membranes in the trans-Golgi network, endosomes and tubular vesicles (GSVs) (Coster et al., 2004; Govers et al., 2004). Upon stimulation, either by insulin or contraction, GLUT4 is transported to the plasma membrane and facilitates glucose uptake into the cell. The majority of glucose uptake in insulin sensitive tissues is mediated by GLUT4, and as such, it plays a pivotal role in mediating whole body glucose homeostasis. Alterations in GLUT4 protein and plasma membrane content have been associated with various metabolic pathologies, including type two diabetes and obesity.

GLUT4 has three cysteine residues and was found to be palmitoylated at Cys223 in 3T3 adipocytes (Ren et al., 2015). Following a substitution of Cys223 with a serine residue, GLUT4 palmitoylation, insulin mediated trafficking and glucose uptake were ablated (Ren et al., 2015). DHHC7 was later identified as the palmitoytransferase responsible for its palmitoylation and subsequent trafficking (Du et al., 2017). Furthermore, DHHC7 silencing in fibroblast-like Chinese hamster ovary cells transfected with a human insulin receptor (CHO-IR) saw suppressed insulin-mediated GLUT4 trafficking to the plasma membrane, further confirming the role of GLUT4 palmitoylation (through DHHC7) regulating glucose uptake (Du et al., 2017). Interestingly, DHHC7-KO mice display hyperglycemia and impaired glucose tolerance, key hallmarks of type two diabetes (Du et al., 2017). Collectively, these results suggest that GLUT4 palmitoylation through DHHC7 is important for GLUT4 trafficking and resultant glucose uptake.

#### 3.2.2 GLUT1

GLUT1 has six cysteine residues, with several located on the cytoplasmic surface of the plasma membrane and was first identified as palmitoylated in blood brain barrier capillaries of rats, using ^3^H palmitate labeling (Pouliot and Béliveau, 1995). Similarly, in both STZ-induced diabetic and diet-induced hyperglycemic rats, enhanced GLUT1 palmitoylation was observed in the blood brain barrier capillaries, suggesting palmitoylation may regulate glucose transport activity in hyperglycemia (Pouliot and Béliveau, 1995). Furthermore, elevated GLUT1 palmitoylation and cerebral glucose uptake was additionally observed in the human peripheral blood mononuclear cells following treatment with the selective serotonin reuptake inhibitor fluoxetine (Stapel et al., 2019). More recently, in glioblastoma (GBM) cells lines, GLUT1 was found to be palmitoylated on Cys209 and the palmitoyltransferase responsible for its palmitoylation was DHHC9 (Zhang et al., 2021). This work also demonstrated that palmitoylation by DHHC9 was imperative for GLUT1 maintenance on the plasma membrane (Zhang et al., 2021). Substitution of Cys209 for a serine residue resulted in decreased glycolysis and subsequent GBM tumour growth, indicating that GLUT1 palmitoylation by DHHC9 is involved in promoting GBM tumorigenesis through upregulating glycolytic processes (Zhang et al., 2021).

All in all, it appears that GLUT4 and GLUT1 palmitoylation may be involved in regulating glucose uptake in various tissues. Given the importance of these two membrane substrate transporters in modulating glucose homeostasis, further work investigating their palmitoylation status in highly metabolic tissue such as skeletal and cardiac tissue is warranted.

## 4 Kinases that regulate plasma membrane transport protein trafficking

Signalling cascades that regulate cellular metabolism are often dependent on protein kinase signalling. AMP-activated protein kinase (AMPK) and serine/threonine kinase Akt are ubiquitously expressed central homeostatic regulators involved in orchestrating signalling pathways that profoundly influencing metabolic control. Upon activation, largely through phosphorylation, Akt and AMPK are major effectors of insulin and non-insulin induced signalling cascades, respectfully, resulting in the trafficking of membrane substrate transporters to the plasma membrane. As their correct functioning is imperative to metabolic homeostasis, further understanding of their regulatory mechanisms is warranted.

### 4.1 AMPK

In response to a decrease in cellular ATP and a subsequent increase in AMP, AMPK is phosphorylated on threonine residue 172 (Thr-172), which is located in the activation loop of the kinase, by upstream kinases such as LKB1 (liver kinase B1). AMPK is a heterotrimeric enzyme complex that comprises of a catalytic α-subunit with β- and γ- regulatory subunits. It’s known to localize to various intracellular compartments including the plasma membrane, lysosomes and mitochondria. The activation of AMPK has a variety of metabolic consequences, including the recruitment of GLUT4 and CD36 to the plasma membrane following the phosphorylation and inactivation of the Rab GTPase proteins TBC1D1/4 (Chavez et al., 2008; Samovski et al., 2012; Whitfield et al., 2017). Consequently, activation of AMPK increases glucose uptake and fatty acid uptake, while suppressing synthesis of fatty acids, cholesterol, and proteins. While phosphorylation has historically been the most well understood PTM regulating AMPK function and activity, the subunits of AMPK have been observed to undergo lipid modifications (Warden et al., 2001; Ren et al., 2013).

A proteomic analysis of palmitoylated proteins in adipocytes observed that AMPKα was palmitoylated (Ren et al., 2013). Later work in cells with serine substitutions found that AMPKα was palmitoylated on Cys209 and Cys543 (Sun and Du, 2022), and this was further demonstrated to be involved in regulating its localization and tethering it to membranes (Sun and Du, 2022). Moreover, inhibition of AMPKα palmitoylation blunted AMPK phosphorylation at Thr172 (Sun and Du, 2022). Therefore, this work suggests that AMPKα palmitoylation is paramount to the ability of AMPK to be phosphorylated and implicates palmitoylation in the regulation of AMPK activity (Sun and Du, 2022). With co-immunoprecipitation assays, DHHC17 was identified as the palmitoyltransferase inducing AMPKα palmitoylation. Following DHHC17 inhibition, AMPK phosphorylation was ablated and mouse hepatic glucose metabolism was impaired (Sun and Du, 2022). These findings highlight that AMPKα palmitoylation by DHHC17 is required for AMPK palmitoylation and subsequent phosphorylation. Given the well-established role of AMPK in mediating plasma membrane transport proteins, it would be of great interest to uncover the functional consequences of AMPKα palmitoylation, to better comprehend how lipid modifications mediate the acute response to energetic stress.

### 4.2 Akt

Akt is recruited to the plasma membrane from the cytosol by activated phosphoinositide 3- kinase (P13K) which converts phosphatidylinositol 4,5- bisphosphate (PIP2) to phosphatidylinositol 3, 4, 5- triphosphate (PIP3) (Alessi et al., 1997). While localized on the plasma membrane, Akt is well known to be regulated through its phosphorylation by PDK1 and mTORC2. Akt is involved in the insulin signalling cascade resulting in the phosphorylation of AS160 and subsequent translocation of GLUT4 and CD36 to the plasma membrane. Membrane attachment of Akt is crucial to its activation, and given the ability of palmitoylation to affect membrane association and tethering, it could be conceptualized that palmitoylation mediates this process.

Recent work in macrophages reported palmitoylation of Akt (Xiong et al., 2021). Using a non-specific pharmacological inhibitor of palmitoylation, 2-bromopalmitate (2-BP), Akt palmitoylation, and membrane distribution were suppressed. Additionally, the inhibition of Akt palmitoylation blunted its phosphorylation (Xiong et al., 2021). Using flag-Akt plasmids and serine substitutions, Cys60 was identified to be the palmitoylated cysteine residue on Akt (Xiong et al., 2021). Furthermore, inhibition of fatty acid synthase (FASN), which is involved in *de novo* fatty synthesis of palmitate, also impaired Akt palmitoylation providing insight into the source of the palmitoyl-CoA driving palmitoylation (Xiong et al., 2021). Interestingly, in LPS-stimulated macrophages treated with metformin, Akt palmitoylation was found to be disrupted as a result of inhibited FASN and this phenomena was concluded to be part of the mechanism behind the anti-inflammatory features of metformin (Xiong et al., 2021). The DHHCs responsible for Akt palmitoylation and potential APTs involved in depalmitoylation are still unknown. Additionally, the function of Akt palmitoylation as a mediator of cellular metabolism warrants further investigation.

## 5 Mitochondrial proteins as targets for palmitoylation

**FIGURE 2:**
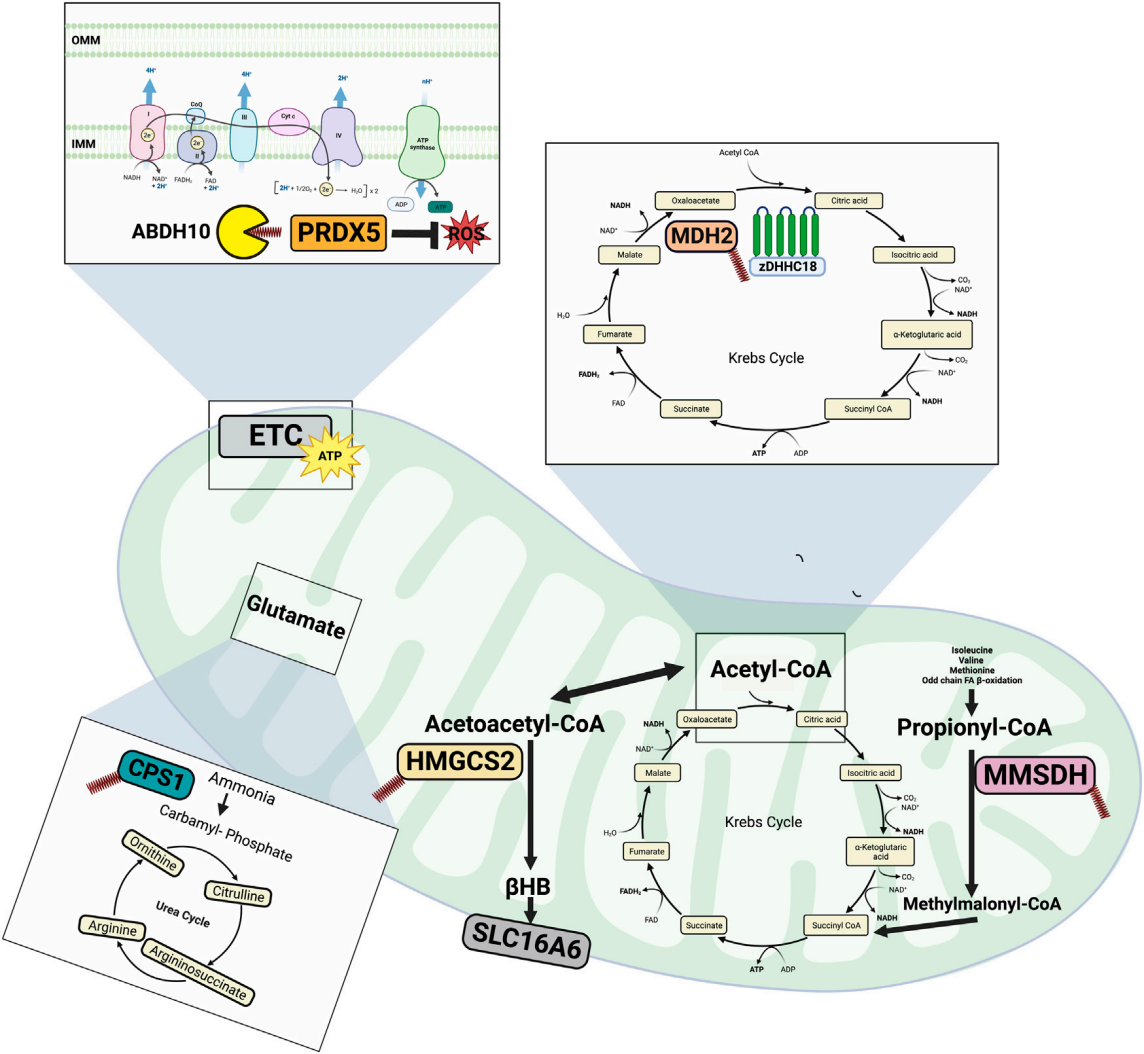
Overview of mitochondrial protein palmitoylation. CPS1 palmitoylation inhibits its catalytic activity thereby reducing amino acid degradation and urea synthesis during starvation. Through depalmitoylation by ABDH10, PRDHX5 regulates mitochondrial antioxidant ability. HMGCS2 is involved in generating ketones (βHB) and its palmitoylation has been implicated in mediating its interaction with PPARα, suggesting a role for palmitoylation in regulating transcription. MDH2 is an important Krebs cycle enzyme and its activity is enhanced following its palmitoylation by zDHHC18. MMSDH is part of the distal portions of the valine and pyrimidine pathway and has been observed to undergo spontaneous autopalmitoylation in response to mitochondrial levels of long chain fatty acyl-CoAs. Made on biorender.com.

In addition to the plasma membrane, cellular metabolism is regulated at the level of the mitochondria. The mitochondrion is characterized by its double membrane, the outer membrane (OMM) is capable of freely transporting ions and small molecules, whereas the inner mitochondrial membrane (IMM) is highly impermeable requiring specific transporters. As the IMM is impermeable to LCACoAs, in order to be taken up into the mitochondria, LCACoAs are converted to acyl carnitine moieties by carnitine palmitoyltransferase I (CPT1) and then transport across the IMM is mediated by carnitine-acylcarnitine translocase. The LCACoAs are then regenerated in the mitochondrial matrix by carnitine palmitoyltransferase II (CPTII). The mitochondria contains 95% of the total CoA pool and is therefore a site of high concentrations of palmitoyl-CoA (Idell-Wenger et al., 1978). Given the importance of the mitochondria in substrate metabolism and the increased concentration of palmitoyl-CoA, the lipid mediated PTM palmitoylation may be a relevant mechanism regulating mitochondrial metabolism. Historically, most of the work in the field of palmitoylation has focused on targets in the ER, Golgi and plasma membrane, while less was known surrounding the role of palmitoylation at the level of the mitochondria. However, palmitoylation has emerged as a potential regulator of mitochondrial metabolic processes. Early pioneering studies investigating the attachment of long chain fatty acids to proteins identified this occurrence on mitochondrial targets, mainly localized in the matrix (Stuckis et al., 1989; Berthiaumes et al., 1994). More recently, large scale proteomics studies have identified potentially hundreds of palmitoylated mitochondrial proteins and their regulatory enzymes (Kostiuk et al., 2008; Kostiuk et al., 2009), however only a handful of the identified proteins have had their palmitoylation status confirmed and the function of their palmitoylation characterized. This section will therefore explore mitochondrial proteins involved with metabolism that have had their palmitoylation status confirmed, which are outlined in Figure 2.

### 5.1 The first palmitoylated mitochondrial proteins: Methylmalonyl semialdehyde dehydrogenase (MMSDH) and carbamoyl-phosphate synthetase 1 (CPS 1)

The first confirmed and characterized palmitoylated mitochondrial enzymes were MMSDH (Berthiaume et al., 1994) and CPS 1 (Corvi et al., 2001). MMSDH is located in the mitochondrial matrix and is part of the distal portions of the valine and pyrimidine pathways. It catalyzes the oxidative decarboxylation of malonate semialdehyde to acetyl-CoA and methylmalonate semialdehyde to propionyl-CoA. Experiments from bovine livers determined that MMSDH activity was inhibited by long chain fatty acids, suggesting a novel mechanism of regulation by lipid PTMs (Deichaite et al., 1993). Furthermore, the fatty acylation of MMSDH only required acyl-CoA and occurred spontaneously *in vitro*, suggesting that mitochondrial levels of long chain fatty acyl-CoAs may regulate fatty acylation of MMSDH. Biochemical and mutagenesis evidence then implicated Cys319 as the target of fatty acylation on MMSDH (Berthiaume et al., 1994). In addition to MMSDH, another mitochondrial dehydrogenase, glutamate dehydrogenase, was predicted to be palmitoylated through the same spontaneous mechanism as MMSDH (Berthiaume et al., 1994). Following the identification of MMSHD as being regulated by lipid PTMs, CPS 1 was observed to be palmitoylated in rat liver, inhibiting its catalytic activity (Corvi et al., 2001). CPS 1 is the first and rate limiting step of urea synthesis, and removes the by-product of amino acid catabolism, ammonia. Following the modification of CPS1 active site cysteine residues, palmitoylation was blunted, indicating that CPS 1 is palmitoylated on at least one cysteine residue (Corvi et al., 2001). The function of CPS 1 palmitoylation was hypothesized to reduce amino acid degradation and urea secretion in order to spare nitrogen during starvation, a time when FA oxidation in the mitochondria is high (Corvi et al., 2001). As both MMSDH and CPS 1 activity is regulated by palmitoylation, consequently, mitochondrial palmitoylation has been postulated to facilitate metabolic crosstalk between the pathways involved in fatty acid and amino acid oxidation (Corvi et al., 2001).

### 5.2 Mitochondrial HMG-CoA synthase (HMGCS2)

HMGCS2 is a mitochondrial matrix enzyme that catalyzes the rate limiting step for ketogenesis, and plays an important role in lipid metabolism. Within the mitochondria, HMGCS2 catalyzes the condensation of acetyl-CoA with acetoacetyl-CoA to form 3-hydroxy-3-methylglutaryl-CoA (HMG-CoA) and CoA, which is then converted to ketone bodies during fasting or starvation. HMGCS2 was first identified as a palmitoylated mitochondrial protein in large scale proteomics screens. Later studies observed that the palmitoylation of HMCGS2 occurred spontaneously in a palmitoyl-CoA dependent fashion, as the *in vitro* experiments contained only the purified enzyme, buffer and varying concentrations of palmitoyl-CoA (Kostiuk et al., 2008). HMGCS contains nine cysteine residues and two cysteines, Cys166 and Cys305, were found to be palmitoylated. Interestingly, it was observed Cys305 was the main acylated cysteine residue, whereas Cys166 acts as a palmitoyl donor through a transacylation mechanism, a mechanism termed catalytic cysteine palmitoyl relay (CCPR) (Kostiuk et al., 2008). Later work found that the palmitoylation of HMGCS2 was found to be required for its well characterized interaction with the transcription factor peroxisome proliferator-activated receptor alpha (PPARα) (Kostiuk et al., 2010). It is important to note these findings are the first to demonstrate that mitochondrial palmitoylation may be a novel modulator of transcription, suggesting that palmitoylation could facilitate fine-tuned responses of transcription levels of certain proteins to fulfill metabolic requirements of the mitochondria.

### 5.3 Malate dehydrogenase 2 (MDH2)

Malate dehydrogenase 2 (MDH2) is a key enzyme in the Krebs cycle and catalyzes the oxidation of malate to oxaloacetate, using NAD^+^ as a cofactor. Interestingly, while investigating its role in ovarian cancer metabolism, MDH2 was found to be palmitoylated on Cys138 resulting in increased activity of the enzyme (Pei et al., 2022). When glutamine was omitted from the cell media, DHHC18 and MDH2 had the greatest interaction, indicating that DHHC18 is able to sense glutamine availability with consequences for palmitoylating MDH2. These results suggest that MDH2, through DHHC18, regulates mitochondrial metabolism *via* glutamine availability in cancer. Furthermore, MDH2 palmitoylation was associated with enhanced mitochondrial respiration to promote ovarian cancer growth (Pei et al., 2022). In further support, patients with high grade serous ovarian cancer had elevated levels of MDH2 palmitoylation and high levels of DHHC18 mRNA (Pei et al., 2022). Importantly, as mentioned above, DHHC18 is the first palmitoyl transferase identified within the mitochondria, and potentially opens up new possibilities of mitochondrial palmitoylation targets. Furthermore, the role of MDH2 palmitoylation in other metabolic diseases, such as diabetes and NAFLD remains to be elucidated.

### 5.4 PRDX5

Aside from ATP production, mitochondria represent a large source of endogenous reactive oxygen species (ROS). PRDX5 is a thioredoxin peroxidase that in the early 2000s was identified within peroxisomes, cytosol and mitochondria. PRDX5 contains three cysteine residues (Cys100, Cys105 and Cys204) and was recently discovered to be palmitoylated on Cys100 (Cao et al., 2019). Work using a pan-APT inhibitor caused an increase in PRDX5 palmitoylation, indicating regulation by mitochondrial depalmitoylases. Employing a depalmitoylase probe and novel spatially constrained mitochondrial APT inhibitor, a previously unknown depalmitoylase ABHD10, was identified as responsible for PRDX5 depalmitoylation (Cao et al., 2019). As such, when ABDH10 was knocked down, PRDX5 palmitoylation was increased whereas in contrast, ABDH10 overexpression decreased PRDX5 palmitoylation (Cao et al., 2019). The same report observed that following the inhibition of APTs with a pan-APT inhibitor, impaired mitochondrial antioxidant capacity was identified in these cells (Cao et al., 2019). Interestingly, palmitoylation of PRDX5 on Cys100 prevents the residue from being in the free thiol form, which is required for its nucleophilic attack on H_2_O_2_ (Cao et al., 2019). Therefore, PRDX5 regulates mitochondrial antioxidant ability through its depalmitoylation by ABDH10 (Cao et al., 2019). These findings suggest that palmitoylation plays a role in the maintenance of mitochondrial redox homeostasis and provides a novel link between protein lipidation and mitochondrial antioxidant buffering capacity. While the depalmitoylase in this process has been identified, further research is required to identify the method by which palmitoyl-CoA is covalently added to PRDX5. It would also be of interest to identify the role of PRDX5 palmitoylation and depalmitoylation in metabolic diseases.

## 6 Conclusion

Collectively, palmitoylation represents an important PTM that not only functions in modulating substrate uptake across plasma membrane through transport proteins and kinases, but is also emerging as playing a vital role in regulating various metabolic aspects of mitochondrial metabolism. Further work is needed to elucidate the regulatory enzymes involved in mitochondrial protein palmitoylation and the functional outcome of palmitoylation of their target proteins. While the role of palmitoylation in orchestrating subcellular trafficking of key membrane substrate transport proteins to the plasma membrane has been well studied in adipocytes and liver, less is known surrounding its role in skeletal and cardiac muscle. Both tissues display tight homeostatic regulation by the same membrane transport proteins and mitochondrial proteins discussed above, suggesting a promising role for palmitoylation in metabolic regulation within these tissues.

## References

[B1] Abrami L. Audagnotto M. Ho S. Jose Marcaida M. Mesquita F. S. Anwar M. U. (2021). Palmitoylated acyl protein thioesterase APT2 deforms membranes to extract substrate acyl chains. Nat. Chem. Biol. 17, 438–447. 10.1038/s41589-021-00753-2 33707782 PMC7610442

[B2] Aicart-Ramos C. Valero R. A. Rodriguez-Crespo I. (2011). Protein palmitoylation and subcellular trafficking. Biochimica Biophysica Acta (BBA) - Biomembr. 1808, 2981–2994. 10.1016/J.BBAMEM.2011.07.009 21819967

[B3] Alessi D. R. James S. R. Downes C. P. Holmes A. B. Gaffney P. R. J. Reese C. B. (1997). Characterization of a 3-phosphoinositide-dependent protein kinase which phosphorylates and activates protein kinase Balpha. Curr. Biol. 7, 261–269. 10.1016/S0960-9822(06)00122-9 9094314

[B4] Berthiaume L. Deichaite I. Peseckis S. Resh M. D. (1994). Regulation of enzymatic activity by active site fatty acylation. A new role for long chain fatty acid acylation of proteins. J. Biol. Chem. 269, 6498–6505. 10.1016/S0021-9258(17)37399-4 8120000

[B5] Bizzozero O. A. Bixler H. A. Pastuszyn A. (2001). Structural determinants influencing the reaction of cysteine-containing peptides with palmitoyl-coenzyme A and other thioesters. Biochimica Biophysica Acta (BBA) - Protein Struct. Mol. Enzym. 1545, 278–288. 10.1016/S0167-4838(00)00291-0 11342053

[B6] Camp L. A. Hofmann S. L. (1993). Purification and properties of a palmitoyl-protein thioesterase that cleaves palmitate from H-Ras. J. Biol. Chem. 268, 22566–22574. 10.1016/S0021-9258(18)41567-0 7901201

[B7] Cao Y. Qiu T. Kathayat R. S. Azizi S. A. Thorne A. K. Ahn D. (2019). ABHD10 is an S-depalmitoylase affecting redox homeostasis through peroxiredoxin-5. Nat. Chem. Biol. 15, 1232–1240. 10.1038/S41589-019-0399-Y 31740833 PMC6871660

[B8] Chamberlain L. H. Shipston M. J. (2015). The Physiology of protein S-acylation. Physiol. Rev. 95, 341–376. 10.1152/PHYSREV.00032.2014 25834228 PMC4551212

[B9] Chavez J. A. Roach W. G. Keller S. R. Lane W. S. Lienhard G. E. (2008). Inhibition of GLUT4 translocation by Tbc1d1, a Rab GTPase-activating protein abundant in skeletal muscle, is partially relieved by AMP-activated protein kinase activation. J. Biol. Chem. 283, 9187–9195. 10.1074/jbc.M708934200 18258599 PMC2431020

[B10] Chen B. Sun Y. Niu J. Jarugumilli G. K. Wu X. (2018). Protein lipidation in cell signaling and diseases: Function, regulation and therapeutic opportunities. Cell Chem. Biol. 25, 817–831. 10.1016/J.CHEMBIOL.2018.05.003 29861273 PMC6054547

[B11] Corvi M. M. Soltys C. L. M. Berthiaume L. G. (2001). Regulation of mitochondrial carbamoyl-phosphate synthetase 1 activity by active site fatty acylation. J. Biol. Chem. 276, 45704–45712. 10.1074/JBC.M102766200 11577071

[B12] Coster A. C. F. Govers R. James D. E. (2004). Insulin stimulates the entry of GLUT4 into the endosomal recycling pathway by a quantal mechanism. Traffic 5, 763–771. 10.1111/J.1600-0854.2004.00218.X 15355512

[B13] De I. Sadhukhan S. (2018). Emerging roles of DHHC-mediated protein S-palmitoylation in physiological and pathophysiological context. Eur. J. Cell Biol. 97, 319–338. 10.1016/J.EJCB.2018.03.005 29602512

[B14] Deichaite I. Berthiaume L. Peseckis S. M. Patton W. F. Resh M. D. (1993). Novel use of an iodo-myristyl-CoA analog identifies a semialdehyde dehydrogenase in bovine liver. J. Biol. Chem. 268, 13738–13747. 10.1016/s0021-9258(18)86919-8 8514806

[B15] DeMar J. C. Anderson R. E. (1997). Identification and quantitation of the fatty acids composing the CoA ester pool of bovine retina, heart, and liver. J. Biol. Chem. 272, 31362–31368. 10.1074/JBC.272.50.31362 9395466

[B16] Du K. Murakami S. Sun Y. Kilpatrick C. L. Luscher B. (2017). DHHC7 palmitoylates glucose transporter 4 (Glut4) and regulates Glut4 membrane translocation. J. Biol. Chem. 292, 2979–2991. 10.1074/jbc.M116.747139 28057756 PMC5314192

[B17] Duncan J. A. Gilman A. G. (1998). A cytoplasmic acyl-protein thioesterase that removes palmitate from G protein α subunits and p21(RAS). J. Biol. Chem. 273, 15830–15837. 10.1074/jbc.273.25.15830 9624183

[B18] Duncan J. A. Gilman A. G. (1996). Autoacylation of G protein alpha subunits. J. Biol. Chem. 271, 23594–23600. 10.1074/jbc.271.38.23594 8798571

[B19] Folch J. Lees M. (1951). Proteolipides, a new type of tissue lipoproteins; their isolation from brain. J. Biol. Chem. 191, 807–817. 10.1016/S0021-9258(18)55985-8 14861226

[B20] Glatz J. F. C. Nabben M. Heather L. C. Bonen A. Luiken J. J. F. P. (2016). Regulation of the subcellular trafficking of CD36, a major determinant of cardiac fatty acid utilization. Biochimica Biophysica Acta (BBA) - Mol. Cell Biol. Lipids 1861, 1461–1471. 10.1016/J.BBALIP.2016.04.008 27090938

[B21] Govers R. Coster A. C. F. James D. E. (2004). Insulin increases cell surface GLUT4 levels by dose dependently discharging GLUT4 into a cell surface recycling pathway. Mol. Cell Biol. 24, 6456–6466. 10.1128/MCB.24.14.6456-6466.2004 15226445 PMC434240

[B22] Greaves J. Prescott G. R. Gorleku O. A. Chamberlain L. H. (2009). The fat controller: Roles of palmitoylation in intracellular protein trafficking and targeting to membrane microdomains (review). Mol. Membr. Biol. 26, 67–79. 10.1080/09687680802620351 19115144

[B23] Greenwalt D. Lipsky R. Ockenhouse C. Ikeda H. Tandon N. Jamieson G. (1992). Membrane glycoprotein CD36: A review of its roles in adherence, signal transduction, and transfusion medicine. Blood 80, 1105–1115. 10.1182/BLOOD.V80.5.1105.1105 1381234

[B24] Hao J. W. Wang J. Guo H. Zhao Y. Y. Sun H. H. Li Y. F. (2020). CD36 facilitates fatty acid uptake by dynamic palmitoylation-regulated endocytosis. Nat. Commun. 11, 4765. 10.1038/s41467-020-18565-8 32958780 PMC7505845

[B25] Hruz P. W. Mueckler M. M. (2001). Structural analysis of the GLUT1 facilitative glucose transporter (review). Mol. Membr. Biol. 18, 183–193. 10.1080/09687680110072140 11681785

[B26] Idell-Wenger J. A. Grotyohann L. W. Neely J. R. (1978). Coenzyme A and carnitine distribution in normal and ischemic hearts. J. Biol. Chem. 253, 4310–4318. 10.1016/s0021-9258(17)34721-x 207696

[B27] Iwanaga T. Tsutsumi R. Noritake J. Fukata Y. Fukata M. (2009). Dynamic protein palmitoylation in cellular signaling. Prog. Lipid Res. 48, 117–127. 10.1016/J.PLIPRES.2009.02.001 19233228

[B28] Jansen M. Beaumelle B. (2022). How palmitoylation affects trafficking and signaling of membrane receptors. Biol. Cell 114, 61–72. 10.1111/BOC.202100052 34738237

[B29] Jochen’ A. Hays J. Jochen A. (1993). Purification of the major substrate for palmitoylation in rat adipocytes: N-Terminal homology with CD36 and evidence for cell surface acylation. J. Lipid Res. 34, 1783–1792. 10.1016/S0022-2275(20)35741-2 7504047

[B30] Kathayat R. S. Cao Y. Elvira P. D. Sandoz P. A. Zaballa M. E. Springer M. Z. (2018). Active and dynamic mitochondrial S-depalmitoylation revealed by targeted fluorescent probes. Nat. Commun. 9 (9), 334. 10.1038/s41467-017-02655-1 29362370 PMC5780395

[B31] Kerr M. Dennis K. M. J. H. Carr C. A. Fuller W. Berridge G. Rohling S. (2021). Diabetic mitochondria are resistant to palmitoyl CoA inhibition of respiration, which is detrimental during ischemia. FASEB J. 35, e21765. 10.1096/FJ.202100394R 34318967 PMC8662312

[B32] Kerr M. Dodd M. S. Heather L. C. (2017). The “Goldilocks zone” of fatty acid metabolism; to ensure that the relationship with cardiac function is just right. Clin. Sci. 131, 2079–2094. 10.1042/CS20160671 28739841

[B33] Kim C. Lee S. E. Kim S. K. Jang H.-D. Kim Y.-C. Hwang I. (2019). Toll-like receptor mediated inflammation requires FASN-dependent MYD88 palmitoylation. Nat. Chem. Biol. 15, 907–916. 10.1038/s41589-019-0344-0 31427815

[B34] Kleuss C. Krause E. (2003). Galpha(s) is palmitoylated at the N-terminal glycine. EMBO J. 22, 826–832. 10.1093/EMBOJ/CDG095 12574119 PMC145455

[B35] Kong E. Peng S. Chandra G. Sarkar C. Zhang Z. Bagh M. B. (2013). Dynamic palmitoylation links cytosol-membrane shuttling of acyl-protein thioesterase-1 and acyl-protein thioesterase-2 with that of proto-oncogene H-ras product and growth-associated protein-43. J. Biol. Chem. 288, 9112–9125. 10.1074/JBC.M112.421073 23396970 PMC3610984

[B36] Kostiuk M. A. Corvi M. M. Keller B. O. Plummer G. Prescher J. A. Hangauer M. J. (2008). Identification of palmitoylated mitochondrial proteins using a bio-orthogonal azido-palmitate analogue. Fed. Am. Soc. Exp. Biol. 22, 721–732. 10.1096/FJ.07-9199COM 17971398 PMC2860959

[B37] Kostiuk M. A. Keller B. O. Berthiaume L. G. (2009). Non-radioactive detection of palmitoylated mitochondrial proteins using an azido-palmitate analogue. Methods Enzymol. 457, 149–165. 10.1016/S0076-6879(09)05009-5 19426867

[B38] Kostiuk M. A. Keller B. O. Berthiaume L. G. (2010). Palmitoylation of ketogenic enzyme HMGCS2 enhances its interaction with PPARalpha and transcription at the Hmgcs2 PPRE. FASEB J. 24, 1914–1924. 10.1096/fj.09-149765 20124434 PMC2874477

[B39] Lefort N. Yi Z. Bowen B. Glancy B. de Filippis E. A. Mapes R. (2009). Proteome profile of functional mitochondria from human skeletal muscle using one-dimensional gel electrophoresis and HPLC-ESI-MS/MS. J. Proteomics 72, 1046–1060. 10.1016/J.JPROT.2009.06.011 19567276 PMC2774790

[B40] Li Q. Velde C. V. Israelson A. Xie J. Bailey A. O. Dong M. Q. (2010). ALS-linked mutant superoxide dismutase 1 (SOD1) alters mitochondrial protein composition and decreases protein import. Proc. Natl. Acad. Sci. U. S. A. 107, 21146–21151. 10.1073/pnas.1014862107 21078990 PMC3000256

[B41] Liang X. Lu Y. Neubert T. A. Resh M. D. (2002). Mass spectrometric analysis of GAP-43/neuromodulin reveals the presence of a variety of fatty acylated species. J. Biol. Chem. 277, 33032–33040. 10.1074/jbc.M204607200 12105219

[B42] Lisanti M. P. Soberer P. E. Vidugiriene J. Tang Z. Hermanowski-Vosatka A. Tu Y.-H. (1994). Characterization of caveolin-rich membrane domains isolated from an endothelial-rich source: Implications for human disease. J. Cell Biol. 126, 111–126. 10.1083/jcb.126.1.111 7517942 PMC2120102

[B43] Lobo S. Greentree W. K. Linder M. E. Deschenes R. J. (2002). Identification of a ras palmitoyltransferase in *Saccharomyces cerevisiae* . J. Biol. Chem. 277, 41268–41273. 10.1074/jbc.M206573200 12193598

[B44] Long J. Z. Cravatt B. F. (2011). The metabolic serine hydrolases and their functions in mammalian Physiology and disease. Chem. Rev. 111, 6022–6063. 10.1021/CR200075Y 21696217 PMC3192302

[B45] Luiken J. J. F. P. Schaap F. G. Van Nieuwenhoven F. A. Van Der Vusse G. J. Bonen A. Glatz J. F. C. (1999). Cellular fatty acid transport in heart and skeletal muscle as facilitated by proteins. Lipids 34, S169–S175. 10.1007/BF02562278 10419138

[B46] Main A. Fuller W. (2022). Protein S-palmitoylation: Advances and challenges in studying a therapeutically important lipid modification. FEBS J. 289, 861–882. 10.1111/FEBS.15781 33624421

[B47] Martin B. R. Wang C. Adibekian A. Tully S. E. Cravatt B. F. (2012). Global profiling of dynamic protein palmitoylation. Nat. Methods 9, 84–89. 10.1038/nmeth.1769 PMC3248616 22056678

[B48] Maynard T. M. Meechan D. W. Dudevoir M. L. Gopalakrishna D. Peters A. Z. Heindel C. C. (2008). Mitochondrial localization and function of a subset of 22q11 deletion syndrome candidate genes. Mol. Cell Neurosci. 39, 439–451. 10.1016/J.MCN.2008.07.027 18775783 PMC2729512

[B49] Mitchell D. A. Mitchell G. Ling Y. Budde C. Deschenes R. J. (2010). Mutational analysis of *Saccharomyces cerevisiae* Erf2 reveals a two-step reaction mechanism for protein palmitoylation by DHHC enzymes. J. Biol. Chem. 285, 38104–38114. 10.1074/JBC.M110.169102 20851885 PMC2992244

[B50] Mitchell D. A. Vasudevan A. Linder M. E. Deschenes R. J. (2006). Protein palmitoylation by a family of DHHC protein S-acyltransferases. J. Lipid Res. 47, 1118–1127. 10.1194/JLR.R600007-JLR200 16582420

[B51] Muszbek L. Haramura G. Cluette-Brown J. E. Van Cott E. M. Laposata M. (1999). The pool of fatty acids covalently bound to platelet proteins by thioester linkages can be altered by exogenously supplied fatty acids. Lipids 34, S331–S337. 10.1007/BF02562334 10419194

[B52] Napoli E. Song G. Liu S. Espejo A. Perez C. J. Benavides F. (2017). Zdhhc13-dependent Drp1 S-palmitoylation impacts brain bioenergetics, anxiety, coordination and motor skills. Sci. Rep. 7 (7), 12796. 10.1038/s41598-017-12889-0 29038583 PMC5643561

[B53] Ohno Y. Kihara A. Sano T. Igarashi Y. (2006). Intracellular localization and tissue-specific distribution of human and yeast DHHC cysteine-rich domain-containing proteins. Biochim. Biophys. Acta Mol. Cell Biol. Lipids 1761, 474–483. 10.1016/j.bbalip.2006.03.010 16647879

[B54] Pei X. Li K. Y. Shen Y. Li J. T. Lei M. Z. Fang C. Y. (2022). Palmitoylation of MDH2 by ZDHHC18 activates mitochondrial respiration and accelerates ovarian cancer growth. Sci. China Life Sci. 65, 2017–2030. 10.1007/S11427-021-2048-2 35366151

[B55] Pepino M. Y. Kuda O. Samovski D. Abumrad N. A. (2014). Structure-function of CD36 and importance of fatty acid signal transduction in fat metabolism. Annu. Rev. Nutr. 34, 281–303. 10.1146/annurev-nutr-071812-161220 24850384 PMC4329921

[B56] Pohl J. Ring A. Korkmaz Ü. Ehehalt R. Stremmel W. (2005). FAT/CD36-mediated long-chain fatty acid uptake in adipocytes requires plasma membrane rafts. Mol. Biol. Cell 16, 24–31. 10.1091/MBC.E04-07-0616 15496455 PMC539148

[B57] Pouliot J. F. Béliveau R. (1995). Palmitoylation of the glucose transporter in blood-brain barrier capillaries. BBA - Biomembr. 1234, 191–196. 10.1016/0005-2736(94)00272-Q 7696293

[B58] Qu M. Zhou X. Wang X. Li H. (2021). Lipid-induced S-palmitoylation as a vital regulator of cell signaling and disease development. Int. J. Biol. Sci. 17, 4223–4237. 10.7150/ijbs.64046 34803494 PMC8579454

[B59] Ren W. Jhala U. S. Du K. (2013). Proteomic analysis of protein palmitoylation in adipocytes. Adipocyte 2, 17–28. 10.4161/ADIP.22117 23599907 PMC3627377

[B60] Ren W. Sun Y. Du K. (2015). Glut4 palmitoylation at Cys223 plays a critical role in Glut4 membrane trafficking. Biochem. Biophys. Res. Commun. 460, 709–714. 10.1016/j.bbrc.2015.03.094 25824042 PMC4426028

[B61] Rocks O. Gerauer M. Vartak N. Koch S. Huang Z. P. Pechlivanis M. (2010). The palmitoylation machinery is a spatially organizing system for peripheral membrane proteins. Cell 141, 458–471. 10.1016/J.CELL.2010.04.007 20416930

[B62] Roth A. F. Feng Y. Chen L. Davis N. G. (2002). The yeast DHHC cysteine-rich domain protein Akr1p is a palmitoyl transferase. J. Cell Biol. 159, 23–28. 10.1083/jcb.200206120 12370247 PMC2173492

[B63] Roth A. F. Wan J. Bailey A. O. Sun B. Kuchar J. A. Green W. N. (2006). Global analysis of protein palmitoylation in yeast. Cell 125, 1003–1013. 10.1016/j.cell.2006.03.042 16751107 PMC2246083

[B64] Samovski D. Su X. Xu Y. Abumrad N. A. Stahl P. D. (2012). Insulin and AMPK regulate FA translocase/CD36 plasma membrane recruitment in cardiomyocytes via Rab GAP AS160 and Rab8a Rab GTPase. J. Lipid Res. 53, 709–717. 10.1194/JLR.M023424 22315395 PMC3307647

[B65] Shang S. Liu J. Hua F. (2022). Protein acylation: Mechanisms, biological functions and therapeutic targets. Signal Transduct. Target Ther. 7, 396. 10.1038/s41392-022-01245-y 36577755 PMC9797573

[B66] Shen L. F. Chen Y. J. Liu K. M. Haddad A. N. S. Song I. W. Roan H. Y. (2017). Role of S-palmitoylation by ZDHHC13 in mitochondrial function and metabolism in liver. Sci. Rep. 7 (7), 2182. 10.1038/s41598-017-02159-4 28526873 PMC5438363

[B67] Smotrys J. E. Linder M. E. (2004). Palmitoylation of intracellular signaling proteins: Regulation and function. Annu. Rev. Biochem. 73, 559–587. 10.1146/annurev.biochem.73.011303.073954 15189153

[B68] Soyombo A. A. Hofmann S. L. (1997). Molecular cloning and expression of palmitoyl-protein thioesterase 2 (PPT2), a homolog of lysosomal palmitoyl-protein thioesterase with a distinct substrate specificity. J. Biol. Chem. 272, 27456–27463. 10.1074/JBC.272.43.27456 9341199

[B69] Stapel B. Gorinski N. Gmahl N. Rhein M. Preuss V. Hilfiker-Kleiner D. (2019). Fluoxetine induces glucose uptake and modifies glucose transporter palmitoylation in human peripheral blood mononuclear cells. Expert Opin. Ther. Targets 23, 883–891. 10.1080/14728222.2019.1675639 31637934

[B70] Stuckis J. W. Lehmann L. H. Siege1 E. (1989). Acylation of proteins by myristic acid in isolated mitochondria. J. Biol. Chem. 264, 6376–6380. 10.1016/S0021-9258(18)83359-2 2703494

[B71] Sun Y. Du K. (2022). DHHC17 is a new regulator of AMPK signaling. Mol. Cell Biol. 42, e0013122. 10.1128/mcb.00131-22 35913156 PMC9387237

[B72] Tao N. Wagner S. J. Lublin D. M. (1996). CD36 is palmitoylated on both N- and C-terminal cytoplasmic tails. J. Biol. Chem. 271, 22315–22320. 10.1074/jbc.271.37.22315 8798390

[B73] Thorne R. F. Ralston K. J. de Bock C. E. Mhaidat N. M. Zhang X. D. Boyd A. W. (2010). Palmitoylation of CD36/FAT regulates the rate of its post-transcriptional processing in the endoplasmic reticulum. Biochim. Biophys. Acta Mol. Cell Res. 1803, 1298–1307. 10.1016/j.bbamcr.2010.07.002 20637247

[B74] Tomatis V. M. Trenchi A. Gomez G. A. Daniotti J. L. (2010). Acyl-protein thioesterase 2 catalyzes the deacylation of peripheral membrane-associated GAP-43. PLoS One 5, e15045. 10.1371/JOURNAL.PONE.0015045 21152083 PMC2994833

[B75] van Oort M. M. Drost R. Janßen L. van Doorn J. M. Kerver J. van der Horst D. J. (2014). Each of the four intracellular cysteines of CD36 is essential for insulin- or AMP-activated protein kinase-induced CD36 translocation. Arch. Physiol. Biochem. 120, 40–49. 10.3109/13813455.2013.876049 24377880

[B76] Vartak N. Papke B. Grecco H. E. Rossmannek L. Waldmann H. Hedberg C. (2014). The autodepalmitoylating activity of APT maintains the spatial organization of palmitoylated membrane proteins. Biophys. J. 106, 93–105. 10.1016/J.BPJ.2013.11.024 24411241 PMC3907232

[B77] Veit M. Sachs K. Heckelmann M. Maretzki D. Hofmann K. P. Schmidt M. F. G. (1998). Palmitoylation of rhodopsin with S-protein acyltransferase: Enzyme catalyzed reaction versus autocatalytic acylation. Biochim. Biophys. Acta 1394, 90–98. 10.1016/S0005-2760(98)00097-6 9767130

[B78] Wang J. Hao J.-W. Wang X. Chen S. Liang G. Zhao Correspondence T.-J. (2019). DHHC4 and DHHC5 facilitate fatty acid uptake by palmitoylating and targeting CD36 to the plasma membrane. Cell Rep. 26, 209–221. 10.1016/j.celrep.2018.12.022 30605677

[B79] Warden S. M. Richardson C. O’Donnell J., J. Stapleton D. Kemp B. E. Witters L. A. (2001). Post-translational modifications of the beta-1 subunit of AMP-activated protein kinase affect enzyme activity and cellular localization. Biochem. J. 354, 275–283. 10.1042/0264-6021:3540275 11171104 PMC1221653

[B80] Wedegaertner P. B. Bourne H. R. (1994). Activation and depalmitoylation of Gs alpha. Cell 77, 1063–1070. 10.1016/0092-8674(94)90445-6 7912657

[B81] Whitfield J. Paglialunga S. Smith B. K. Miotto P. M. Simnett G. Robson H. L. (2017). Ablating the protein TBC1D1 impairs Contraction-induced sarcolemmal glucose transporter 4 redistribution but not insulin-mediated responses in rats. J. Biol. Chem. 292, 16653–16664. 10.1074/jbc.M117.806786 28808062 PMC5633127

[B82] Won S. J. Martin B. R. (2018). Temporal profiling establishes a dynamic S-palmitoylation cycle. ACS Chem. Biol. 13, 1560–1568. 10.1021/ACSCHEMBIO.8B00157/SUPPL_FILE/CB8B00157_SI_007 29733200 PMC6192522

[B83] Xiong W. Sun K. Y. Zhu Y. Zhang X. Zhou Y. H. Zou X. (2021). Metformin alleviates inflammation through suppressing FASN-dependent palmitoylation of Akt. Cell Death Dis. 12, 934. 10.1038/s41419-021-04235-0 34642298 PMC8511025

[B84] Zhang J. Li X. Mueller M. Wang Y. Zong C. Deng N. (2008). Systematic characterization of the murine mitochondrial proteome using functionally validated cardiac mitochondria. Proteomics 8, 1564–1575. 10.1002/pmic.200700851 18348319 PMC2799225

[B85] Zhang Z. Li X. Yang F. Chen C. Liu P. Ren Y. (2021). DHHC9-mediated GLUT1 S-palmitoylation promotes glioblastoma glycolysis and tumorigenesis. Nat. Commun. 12, 5872. 10.1038/s41467-021-26180-4 34620861 PMC8497546

[B86] Zhao L. Zhang C. Luo X. Wang P. Zhou W. Zhong S. (2018). CD36 palmitoylation disrupts free fatty acid metabolism and promotes tissue inflammation in non-alcoholic steatohepatitis. J. Hepatol. 69, 705–717. 10.1016/j.jhep.2018.04.006 29705240

